# AIF1 was identified as an up-regulated gene contributing to CSFV Shimen infection in porcine alveolar macrophage 3D4/21 cells

**DOI:** 10.7717/peerj.8543

**Published:** 2020-02-17

**Authors:** Xiaocheng Gong, Xuepeng Li, Xin You, Aoxue Hu, Min Liu, Huimin Yao, Jun He, Xianghan Zhang, Pengbo Ning

**Affiliations:** 1 School of Life Science and Technology, Xidian University, Xi’an, Shaanxi, China; 2 Engineering Research Center of Molecular & Neuroimaging, Ministry of Education, Xi’an, Shaanxi, China

**Keywords:** CSFV Shimen, AIF1, IL6, Porcine alveolar macrophages

## Abstract

Classical swine fever (CSF) is a disease that is characterized by diffuse hemorrhaging, high fever, and high mortality rates. The pro-inflammatory characteristics of allograft inflammatory factor 1 (AIF1) have been well documented; however, insufficient attention has been given to porcine AIF1. In the present study, AIF1 was identified as a key player contributing to CSFV Shimen infection in porcine alveolar macrophage (PAM) 3D4/21 cell line. Our evaluation showed that AIF1 mRNA and protein are expressed at a time-dependent high level in CSFV Shimen-infected PAM 3D4/21 cells. The transcription and translation of IL6 were also significantly upregulated in infected PAM 3D4/21 cells. By utilizing overexpression RNAs approach, we showed that the cellular AIF1 induced an increased IL6 in PAM 3D4/21 cells. Furthermore, silencing of AIF1 suppressed CSFV Shimen-induced IL6 production in PAM 3D4/21 cells and also inhibited CSFV replication, whereas overexpression of recombinant AIF1 was beneficial for the replication of CSFV Shimen and promoting IL6 production in CSFV Shimen-infected PAM 3D4/21 cells. It is suggested CSFV Shimen induced IL6 in PAM 3D4/21 cells via AIF1 activation, which help clarify the AIF1-related inflammatory processes that occur on CSFV Shimen infected macrophages.

## Introduction

Viruses coevolve with their hosts via poorly-understood mechanisms to ensure their survival. The World Organization for Animal Health lists classical swine fever (CSF) as a highly contagious disease that is characterized by high fever, diffuse hemorrhaging, and a high mortality rate (Postel et al., 2017; Lin et al., 2018). Despite the seriousness of this disease, the inflammatory process that occur in the pathogenesis of CSF are not well understood. As shown by the acute organic damage it causes, classical swine fever virus (CSFV) Shimen strain can proliferate efficiently in macrophages, but does not cause the typical cytopathic effects (Ning et al., 2017). The response to CSFV infection remains focused on macrophages recently.

Allograft inflammatory factor 1 (AIF1) is an important inflammation-responsive scaffold protein that is mainly expressed in immunocytes (Zhao, Yan & Chen, 2013). AIF1 mRNA (GenBank accession number U17919) was initially cloned from activated mouse macrophages (Utans et al., 1995). In humans, the *AIF1* gene is located at chromosomal locus 6p21.3 within the major histocompatibility complex (MHC) class III region (Deininger, Meyermann & Schluesener, 2002). The AIF1 protein functions as a key cytokine regulator of immune responses, and it is generally accepted that AIF1 is involved in various pathological inflammatory processes, including allograft rejection and autoimmune lesions (Zhao, Yan & Chen, 2013). AIF1 can be used in clinical settings as an inflammatory marker of acute and chronic organ transplantation rejection. A study of 26 heart transplant patients revealed that the expression of AIF1 is associated with allograft rejection and the development of cardiac allograft vasculopathy (Autieri et al., 2002). Rat orthotopic transplant analysis models also demonstrated that increased expression of AIF1, which plays a role in monitoring increased immunosuppression, indicates aggressive liver allograft rejection (Nagakawa et al., 2015). In addition, immunohistochemical analysis of human nerve biopsies showed that there is higher AIF1 expression in vasculitic neuropathies than in control nerves and non-inflammatory axonal neuropathies (Laura Broglio et al., 2008). Recently, the role of AIF1 in cancer has also been investigated. After macrophages were stimulated with colony-stimulating factor 1, overexpression of AIF1 led to enhanced tumor cell migration and hepatocellular carcinoma progression (Cai et al., 2017).

Although research on AIF1 has gradually increased, the role of this factor in the face of viral infections is little studied, and the mechanism underlying its role in CSFV Shimen strain infection remains unclear. Here, we describe a study that showed upregulated *AIF1* expression in porcine alveolar macrophage (PAM) 3D4/21 cell line during CSFV Shimen infection using digital gene expression (DGE) tag profiling and molecular biological methods. We also assessed the role of IL6, a lymphokine associated with CSFV infection, and presented a mechanistic hypothesis that CSFV Shimen up-regulate AIF1 expression to induce up-expressed IL6, to help us understand AIF1-related inflammation in CSFV Shimen infection.

## Methods

### Study design, virus strain, and cells

DGE analysis was performed to screen for differentially expressed genes in CSFV-infected PAMs based on previously reported high-throughput sequencing data (Ning et al., 2017), which identified *AIF1* as a key gene, and further testing was conducted to confirm its role during infection by CSFV Shimen. The CSFV Shimen strain was obtained from the Control Institute of Veterinary Bioproducts and Pharmaceuticals. The PAM 3D4/21 cell line (American Type Culture Collection: CRL-2843) were seeded at a density of 2 × 10^7^ cells per flask. When the macrophages reached 70–80% confluence, CSFV Shimen was added to the cultures at a multiplicity of infection of 5 TCID50 (Ning et al., 2017). After incubation with the virus at 37 °C and 5% CO_2_ for 1 h, the virus was removed, and the cells were cultured for 0, 12, 24, and 48 h, and RNA or protein extracts from the cultured cells were collected for subsequent detection (Ning et al., 2016).

### Quantitative polymerase chain reaction

To assess the differences in mRNA expression by quantitative polymerase chain reaction (qPCR), total mRNA was extracted from CSFV-infected and uninfected PAMs. Then, complementary DNA (cDNA) was synthesized from the mRNA by using the Transcriptor First Strand cDNA Synthesis Kit (Takara, Dalian, China) according to the manufacturer’s instructions. Finally, changes in the mRNA expression of AIF1, IL6, and β-actin in the experimental groups (CSFV-infected PAMs) were detected by qPCR conducted on an ABI7300 system using SuperReal PreMix Plus (SYBR Green) and the specific oligonucleotide primers shown in Table 1. The two-step thermal cycler program was as follows: 95 °C for 15 min, followed by 40 cycles of 95 °C for 10 s and 63 °C for 30 s. Three replicate experiments were performed for each experimental group, and the Ct value for each sample was calculated and normalized using the 2^−ΔΔCt^ method.

**Table 1:** Oligonucleotides used in the study.

Targets	Sequence (5′ to 3′)
AIF1-sense	TTATGTCCCTGAAACGAATG
AIF1-antisense	CAGAGTAGCTGAAAGTCTCCC
IL6-sense	GATGCTTCCAATCTGGGTTCA
IL6-antisense	CATTTGTGGTGGGGTTAGGG
β-actin-sense	CAAGGACCTCTACGCCAACAC
β-actin-antisense	TGGAGGCGCGATGATCTT
AIF1-p-Si1-sense	GATCCGCAGGAAGAGAGACTCAATGATTCAAGACGTCATTGAGTCTCTCTTCCTGCTTTTTTA
AIF1-p-Si1-antisense	AGCTTAAAAAAGCAGGAAGAGAGACTCAATGACGTCTTGAATCATTGAGTCTCTCTTCCTGCG
AIF1-p-Si2-sense	GATCCGCCAAACCCTGGATTTACAGGTTCAAGACGCCTGTAAATCCAGGGTTTGGCTTTTTTA
AIF1-p-Si2-antisense	AGCTTAAATAAGCCAAACCCTGGATTTACAGGCGTCTTGAACCTGTAAATCCAGGGTTTGGCG
AIF1-p-Si3-sense	GATCCGGAAGAGAGACTCAATGAAATTTCAAGACGATTTCATTGAGTCTCTCTTCCTTTTTTA
AIF1-p-Si3-antisense	AGCTTAAAAAAGGAAGAGAGACTCAATGAAATCGTCTTGAAATTTCATTGAGTCTCTCTTCCG
AIF1-p-SiNC-sense	GATCCGCTTAAACGCATAGTAGGACTTTCAAGACGAGTCCTACTATGCGTTTAAGCTTTTTTA
AIF1-p-SiNC-antisense	AGCTTAAAAAAGCTTAAACGCATAGTAGGACGTCTTGAATGAGTCCTACTATGCGTTTAAGCG
p-AIF1-sense (EcoR I)	CG GAATTC ATGAGCCAAACCCTGGATTTACAGG
p-AIF1-antisense (BamH I )	CG GGATCC TCAGGGCAACTCAGAGATAGCCTTC

### Western blotting

Total protein was extracted from each cell sample, and the protein concentration was measured using the BCA protein assay kit (Cowin Biotech). Equal amounts of protein were separated by 12% sodium dodecyl sulfate-polyacrylamide gel electrophoresis (SDS-PAGE), and the separated proteins were transferred to a polyvinylidene difluoride (PVDF) membrane (Millipore). The membrane was blocked with 5% bovine serum albumin in Tris-buffered saline and Tween 20 (TBST) for 1 h at 25 °C ± 2 °C, and then incubated with specific primary antibodies raised against AIF1 (ab5076; Abcam), IL6 (ab6672; Abcam), CSFV E2 (MssBio, China), and β-actin (Tianjin Sungene Biotech, China). The membranes were washed with TBST solution six times for 5 min each, and then washed in the same manner after incubation with the secondary antibody for 1 h at room temperature. Western blotting images were obtained using a GeneGnome XRQ chemiluminescence detector (Syngene) and quantified using ImageJ software (National Institutes of Health).

### Confocal immunofluorescence microscopy

PAMs infected with CSFV were washed with phosphate-buffered saline (PBS), fixed with acetone/methanol (1:1) at room temperature for 20 min, and permeabilized with 1% Triton X-100 in PBS for approximately 10 min. The samples were washed with PBS three times, and then incubated with a mouse anti-E2 antibody and a rabbit anti-AIF1 or anti-IL6 antibody at room temperature for 1 h, and then incubated with donkey anti-rabbit IgG and donkey anti-mouse IgG for 1 h at room temperature. Finally, the samples were incubated with 4′, 6-diamidino-2-phenylindole (DAPI), and examined using a confocal microscope (LSM510 META, Zeiss, Germany). Image analysis was performed using the operating software provided with the microscope.

### Gene overexpression and RNA interference

A recombinant plasmid overexpressing AIF1 was constructed, and the primers including restriction sites (Table 1) were designed to amplify AIF1 gene according to archived AIF1 nucleotide sequence. The product of PCR was subcloned into mammalian expression vector pEGFP-C1 (YouBio, China). The constructed AIF1 overexpressed plasmids (p-AIF1) and empty plasmids (p-AIF1-NC) were introduced into overnight cultured macrophages in 6-well plates at 37 °C by using turbofect transfection reagent (Thermo Scientific, Waltham, MA, USA). At 12 h post-transfection, cells were treated with different research requirements.

Three pairs of shRNA sequences targeting different nucleotide sites in the porcine *AIF1* gene were designed by using Invitrogen siRNA online design software (https://rnaidesigner.invitrogen.com/rnaiexpress). A BLAST search was performed to ensure that the shRNAs did not have significant sequence homology with other genes. The 63-nt oligonucleotides were annealed and cloned into the BamHI and HindIII sites of the pGenesil-1 vector (Yingrun Biotech. Co., Ltd., China). The shRNA expression vectors were named AIF1-p-Si1, AIF1-p-Si2, and AIF1-p-Si3. A scrambled negative shRNA, which was named AIF1-p-SiNC, was used as a control. All constructs were confirmed by DNA sequencing using the Sanger method, and are listed in Table 1. TurboFect™ transfection reagent was used to transfect AIF1-p-Si1, AIF1-p-Si2, AIF1-p-Si3, and AIF1-p-SiNC into 3D4/21 cells in 6-well plates at 37 °C and 5% CO_2_. After 16–24 h of transfection, fluorescence microscopy was used to observe the effects of shRNA transfection.

### Statistical analysis

Statistical analysis was carried out by one-way ANOVA using IBM SPSS software (https://www.ibm.com/analytics), and *P* values less than 0.05 were considered statistically significant. Experimental data are reported as mean values ± SE (*n* = 3).

## Results

### Upregulation of AIF1 and IL6 transcription following infection by CSFV Shimen

**Figure 1:**
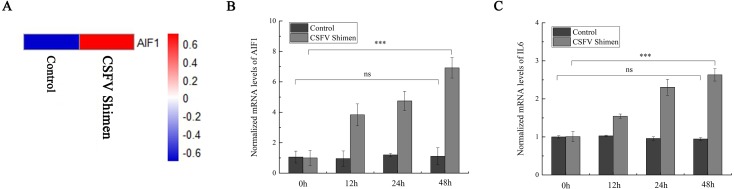
The mRNA expression of allograft inflammatory factor 1 (AIF1) and IL6 in macrophages infected with classical swine fever virus (CSFV) Shimen. (A) Mock and CSFV Shimen infected macrophages were subjected to the digital gene expression tag profiling analysis, and obtained results of AIF1 expression were used to produce a heat map. The red means up-regulated expression of transcription relative to the blue. The analysis revealed that the level of AIF1 expression was strongly associated with CSFV Shimen infection. (B) The expression of AIF1 mRNA was analyzed using quantitative polymerase chain reaction (qPCR) in macrophages infected with CSFV Shimen for 0, 12, 24, and 48 h. (C) qPCR analyzed the expression IL6 mRNA in macrophages infected with CSFV Shimen for 0, 12, 24, and 48 h. We used β-actin as the reference gene.

After the PAMs 3D4/21 cells were infected with CSFV-Shimen for 48 h, the DGE data showed that *AIF1* expression was higher in infected cells than in the control PAMs (Fig. 1A), indicating that CSFV Shimen infection upregulates the transcription level of *AIF1*. Analysis of the basic characteristics of porcine AIF1 is shown in Fig. S1. The qPCR analysis revealed the *AIF1* transcription level was significantly higher in the CSFV Shimen-infected macrophages than in the control (mock infected) macrophages (Fig. 1B). IL6 transcription level was also assessed in CSFV Shimen-infected PAMs to identify the inflammatory responses triggered in response to infection, and a similar trend of increased transcriptional level was observed (Fig. 1C).

### Detection of AIF1 and IL6 protein expression in macrophages following CSFV Shimen infection

**Figure 2:**
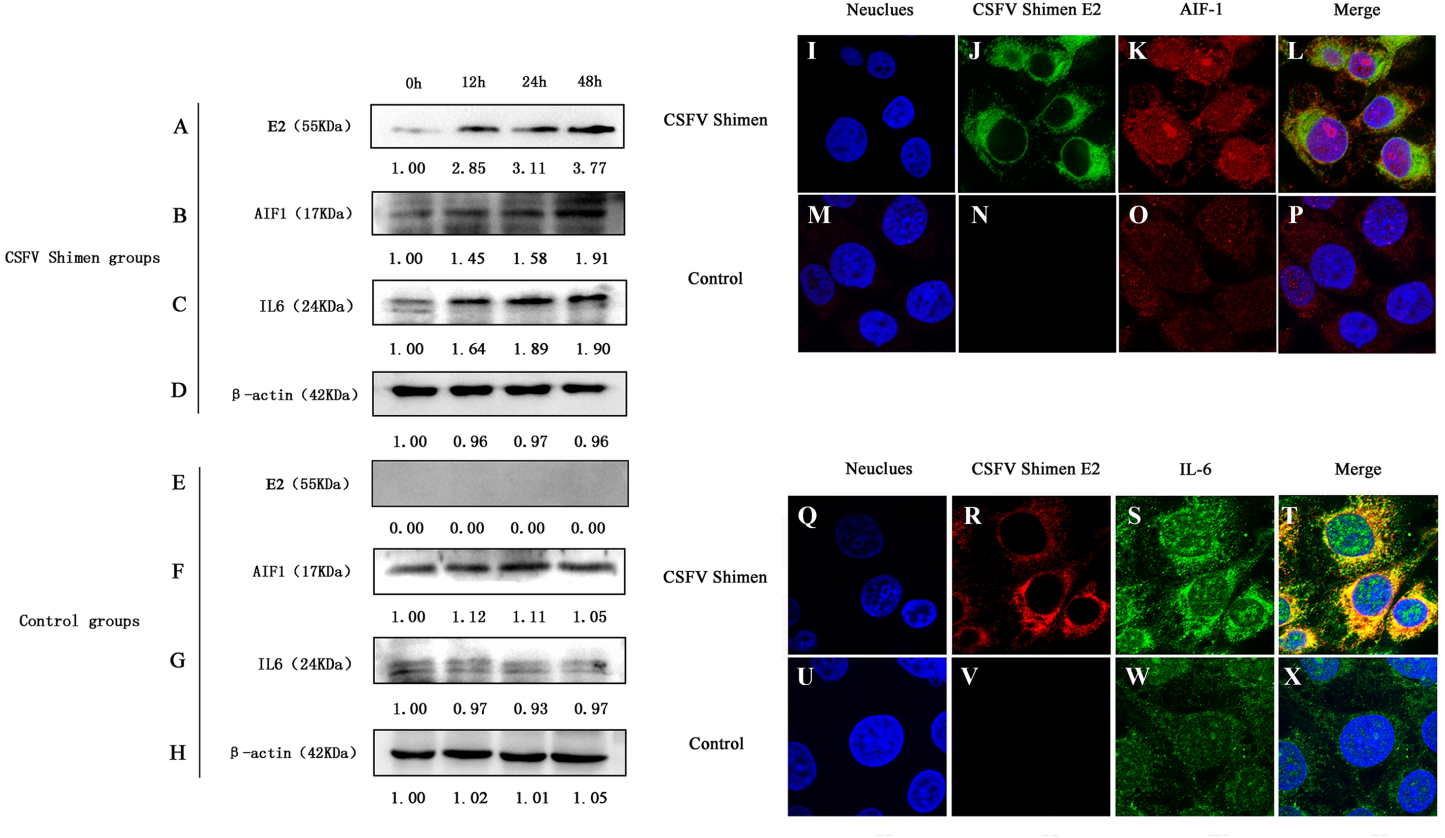
Effects of CSFV Shimen infection on the abundance of allograft inflammatory factor 1 (AIF1) and IL6 protein. The bands of E2 protein, AIF1 protein, IL6 protein, and β-actin protein by western blotting in the CSFV Shimen group are shown in A, B, C, D, respectively, and which in control groups are shown in E, F, G, H, respectively. “0h” was as a start point after 1 h of virus incubation at 37 °C in 5% CO_2_. The values on the top of each band meant relative protein expression level in which the value of “0h” was normalized as “1”, and “0” meant out of protein expression. Neuclues stained by DAPI in CSFV Shimen group and control group are shown in I and M, respectively. The E2 protein and AIF1 protein transiently expressed in macrophages infected with CSFV Shimen group is shown in J and K, respectively, and which expressed in the control group is shown in N and O, respectively. Overlay with DAPI in CSFV Shimen group and control group are shown in L and P, respectively. Neuclues stained by DAPI in CSFV Shimen group and control group are shown in Q and U, respectively. The E2 protein and IL6 protein transiently expressed in macrophages infected with CSFV Shimen group is shown in R and S, respectively and which expressed in the control group is shown in V and W, respectively. Overlay with DAPI in CSFV Shimen group and control group are shown in T and X, respectively.

Western blotting analysis revealed differences in AIF1 and IL6 protein expression levels between CSFV-infected and uninfected control cells, which demonstrated that AIF1 and IL6 levels increased significantly in the first 48 h after infection relative to the levels at the 0 time point (Figs. 2A–2H). Confocal microscopy was used to support the protein expression analysis. The colocalization of AIF1 and E2, an envelope glycoprotein of CSFV (Li et al., 2018), was observed (Figs. 2I–2P). The results of Western blotting quantification by ImageJ software supported that AIF1 and IL6 were significantly more abundant in the infected PAMs than in the uninfected control PAMs (Fig. S2). Meanwhile, the analysis by confocal laser scanning microscopy showed that there was significantly more AIF1 and IL6 in the CSFV Shimen-infected PAMs than in the uninfected control cells (Figs. 2Q–2X). A quantitative analysis was carried out to determine the significance of the confocal analysis (Fig. S3). These findings suggest that AIF1 plays a pro-inflammatory role in PAMs in response to infection by CSFV Shimen.

### AIF1 stimulates PAMs 3D4/21 cells to augment IL6

**Figure 3:**
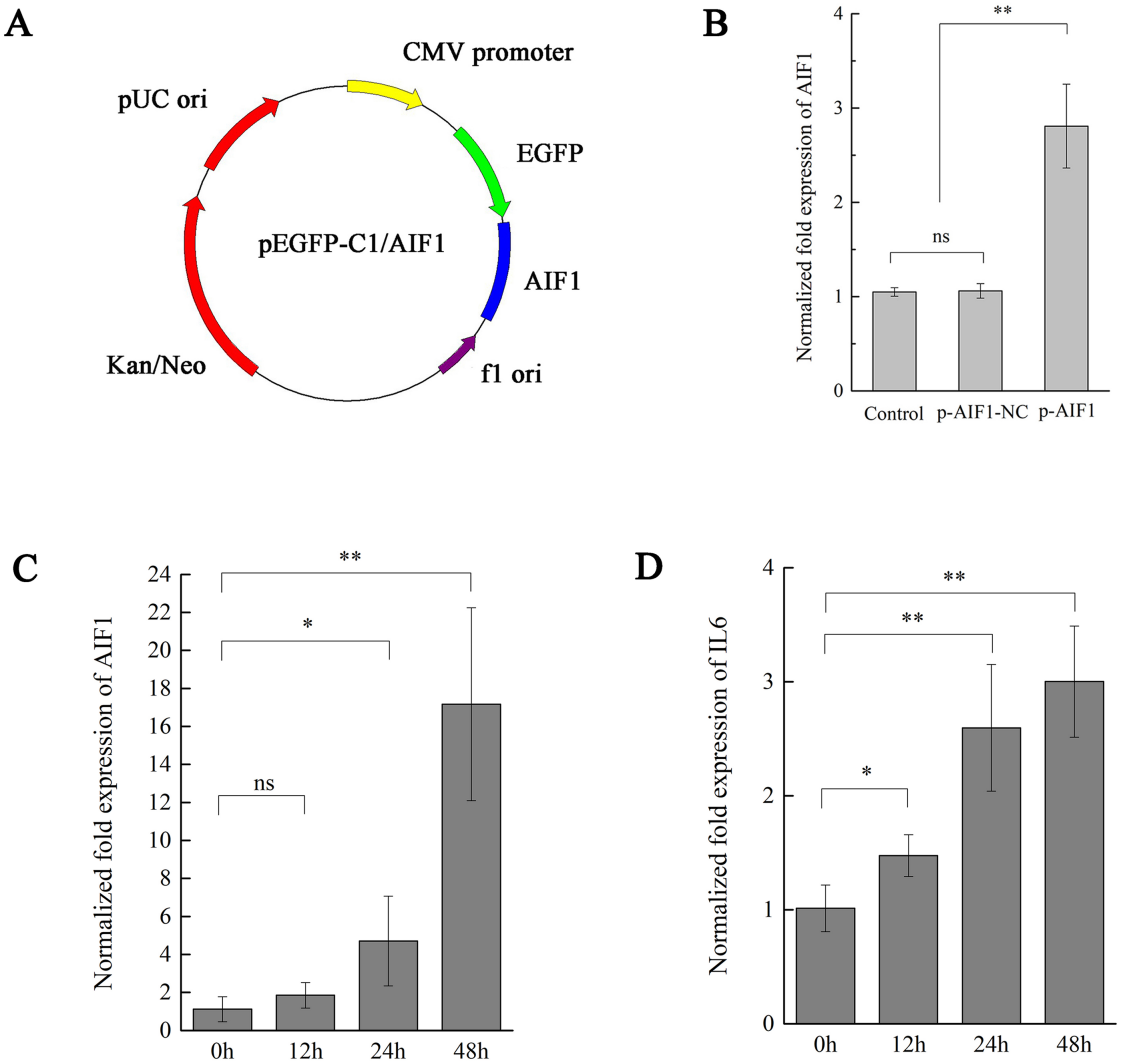
AIF1 stimulates PAMs 3D4/21 cells to augment IL-6 in mRNA levels. (A) Construction and characterization of the pEGFP-C1 vector for overexpression AIF1. (B) qPCR analysis results suggested that p-AIF1 has a significant up-regulation effect on AIF1 expression compared with the control and p-AIF1-SiNC groups. (C) AIF1 expression in macrophages after transfection of p-AIF1. (D) IL6 expression was induced by overexpression of AIF1. The relative values from qPCR analysis were obtained by 2^−ΔΔCt^ method. These results are representative of three independent experiments with consistent conclusion.

We have shown that *CSFV Shimen* upregulated the AIF1 and IL6 expression in PAMs. A previous study showed AIF1 augments production of IL6 by a mouse macrophage line (Watano et al., 2001). To further elucidate the existence of certain association between AIF1 and IL6 levels on CSFV infection in PAMs, we transfected overexpressing AIF1 vectors into PAMs 3D4/21 cells to clarify cause–effect connection between those factors. First, overexpressing AIF1 plasmids (p-AIF1) were constructed (Fig. 3A) to compare its effects on AIF1 expression in PAMs 3D4/21 cells with the control groups (p-AIF1-NC or mock transfectants). Figure 3B showed that the expressing of AIF1 in PAMs 3D4/21 cells were significantly induced by overexpressing AIF1 plasmids (p-AIF1), and this effect was dose-dependent (Fig. 3C). Next, IL6 alterations of PAMs 3D4/21 cells by overexpressing AIF1 were examined, and relative transcription levels of IL6 analysis showed the significantly upregulated IL6 expression in PAMs 3D4/21 cells with the dose-dependent AIF1 expression (Fig. 3D). No significant results were obtained in the control groups (Fig. S4).

### CSFV Shimen induces IL6 expression via AIF1

**Figure 4:**
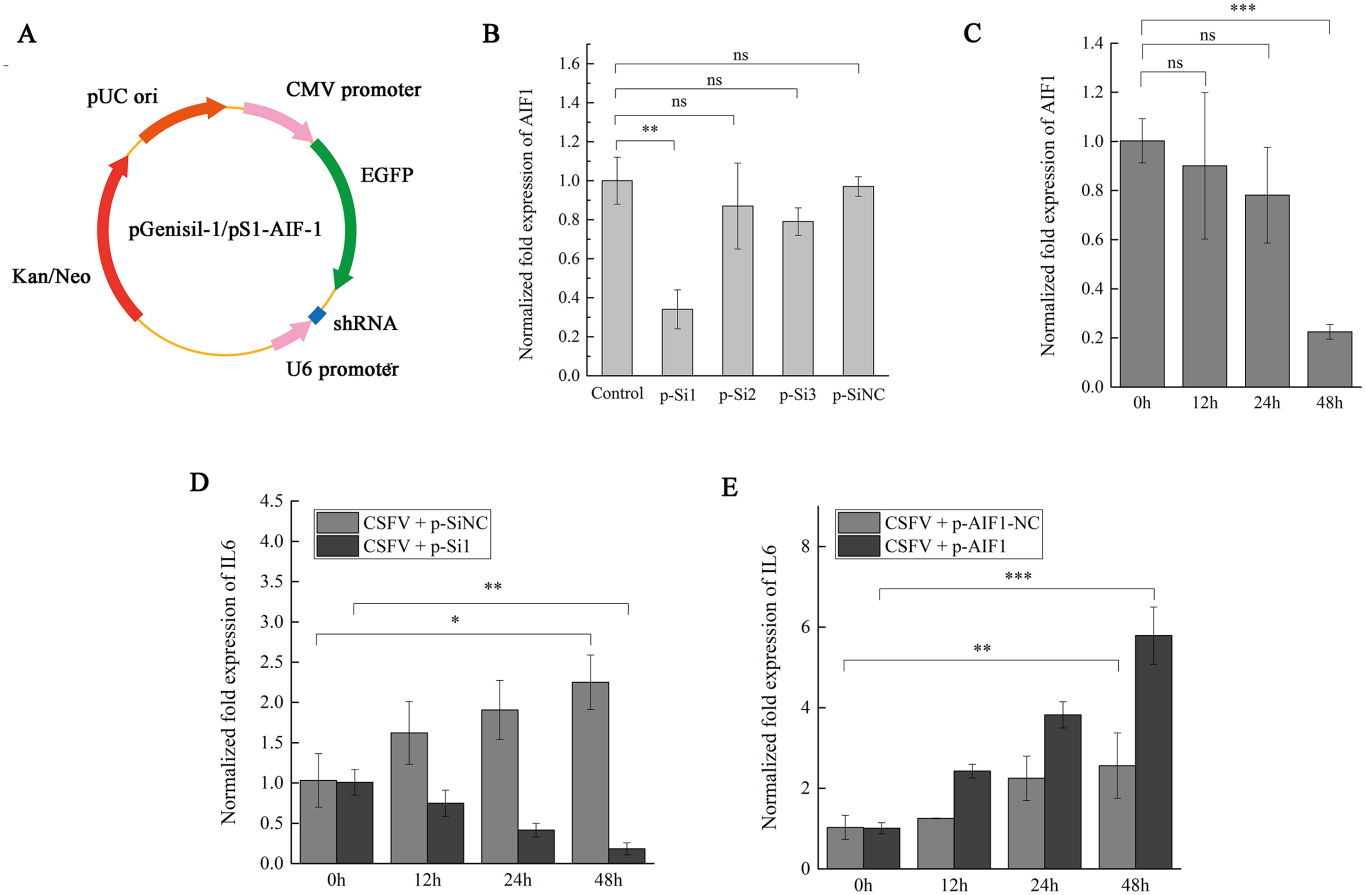
AIF1 expression in CSFV Shimen-infected PAMs 3D4/21 cells is required for an increase of IL6 expression. (A) Construction and characterization of the pGenesil vector for inhibit the expression of AIF1. (B) qPCR analysis results suggested that AIF1-p-Si1 has a significant inhibitory effect on AIF1 expression compared with the control and AIF1-p-SiNC groups. (C) AIF1 expression in PAMs 3D4/21 cells after transfection of AIF1-p-Si1. (D) RNA silencing of AIF1 reduced CSFV Shimen induced-IL6 expression. (E) Overexpression of AIF1 augmented CSFV Shimen induced-IL6 expression. The relative values from qPCR analysis were obtained by 2^−ΔΔCt^ method. These results are representative of three independent experiments with consistent conclusion.

To further observe the impact of *AIF1* on the inflammatory response in CSFV Shimen-infected PAMs 3D4/21 cells, after *AIF1* expression in PAMs was silenced by siRNA, CSFV Shimen was inoculated in PAMs 3D4/21 cells comprised with the control groups to determine whether CSFV Shimen induces IL6 expression via AIF1. As shown is Fig. 4A, siRNA plasmids were constructed to compare the interference effects of three designed interference sequences (Fig. 4A). After the different plasmids were transfected (Fig. S5), PCR (Fig. S5) and qPCR analysis suggested that AIF1-p-Si1 has a significant inhibitory effect on AIF1 expression compared with the control AIF1-p-SiNC-transfected cells (Figs. 4B and 4C). Therefore, we transfected cells with AIF1-p-Si1 and AIF1-p-SiNC plasmids to investigate the role of AIF1 in CSFV Shimen-induced IL6 expression. The results showed that the levels of IL6 were blocked in AIF1-p-Si1-transfected PAMs 3D4/21 cells infected with CSFV Shimen compared to the levels in infected cells transfected with p-SiNC (Fig. 4D). In contrast, through the overexpression of recombinant AIF1 in PAMs, the levels of IL6 were up-regulated in p-AIF1-transfected PAMs 3D4/21 cells infected with CSFV Shimen compared to those transfected with p-AIF1-NC (Fig. 4E).

### AIF1 confers susceptibility to CSFV Shimen in PAMs 3D4/21 cells

**Figure 5:**
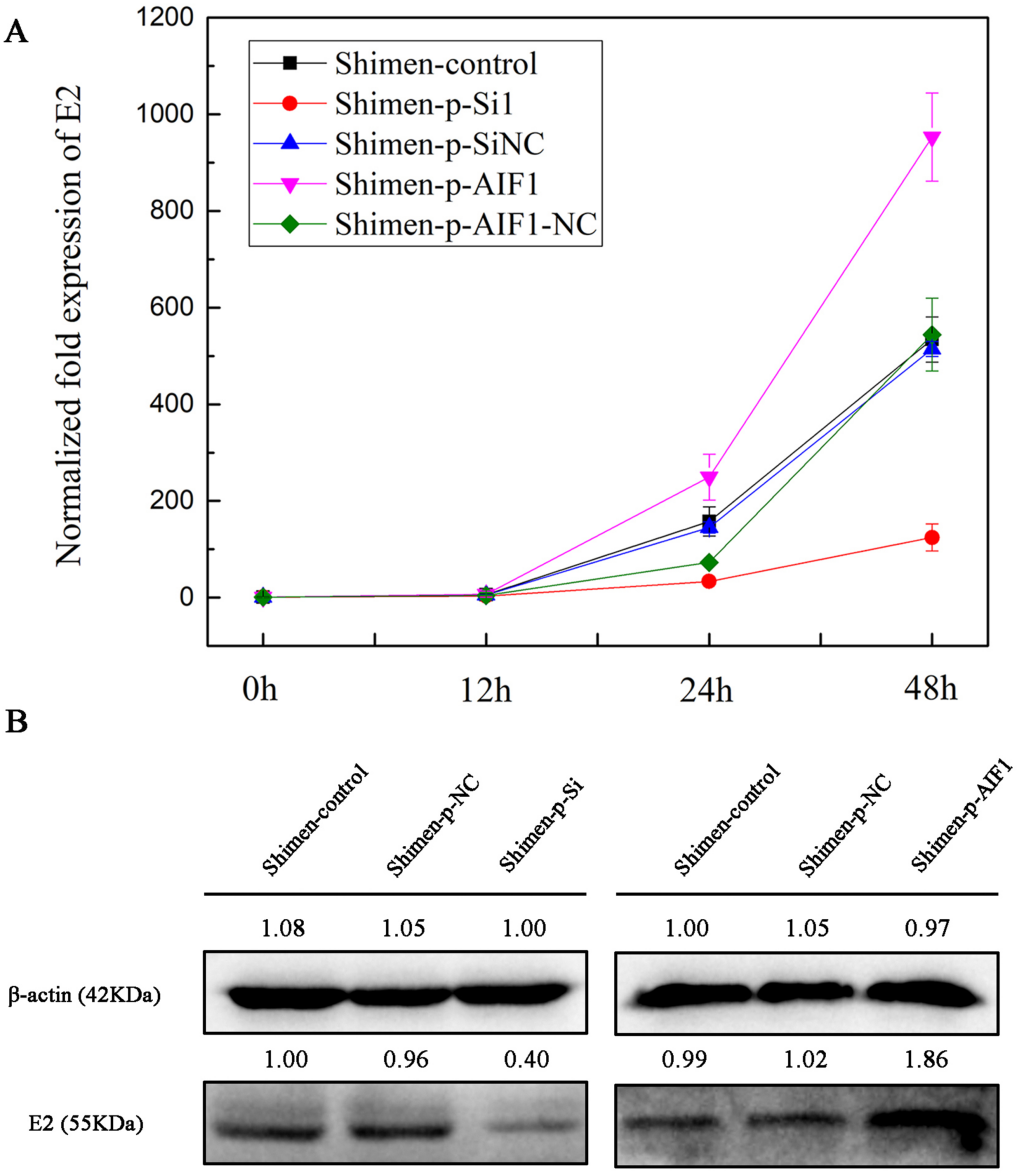
AIF1 confers susceptibility to CSFV Shimen in macrophages. (A) Macrophages transfected with AIF1-p-Si1, AIF1-p-SiNC, p-AIF1 and p-AIF1-NC were infected with CSFV Shimen, respectively. Normal macrophages infected with CSFV Shimen served as the experimental control. Viral replication was determined by qPCR, which showed the expression of CSFV Shimen genome equivalents increased over the 48 h experimental period in the control, AIF1-p-SiNC and p-AIF1-NC groups, In addition, a reduced replication rate of CSFV Shimen was observed in macrophages treated with small interfering RNAs targeting AIF1 whereas a sharp increase at 48 h post-infection was observed in macrophages treated with overexpression AIF1 group (p-AIF1). (B) CSFV Shimen E2 protein expressed in macrophages infected with CSFV Shimen at 48 h post-infection from different groups were compared by western blotting. These results are representative of 3 independent experiments with consistent conclusion.

To investigate the relevance of AIF1 in the CSFV Shimen life cycle, the p-Si1 plasmid was used to target *AIF1* in macrophages and compared to cells treated with a scrambled siRNA (AIF1-p-SiNC) and mock-treated cells before CSFV Shimen were inoculated into PAMs 3D4/21 cells. The qPCR analysis results showed accelerated replication of CSFV Shimen in the AIF1-p-SiNC-treated macrophages compared to a significant inhibition of CSFV Shimen growth in AIF1-p-Si1-treated macrophages, which was consistent with the results of the western blotting analysis (Fig. 5 and Fig. S6). Compare results were demonstrated in Fig. 5 that AIF1 over-expression is beneficial for the replication of CSFV Shimen. Together, these findings highlight a key role for cellular AIF1 in CSFV Shimen growth.

## Discussion

Macrophages are associated with a variety of disorders, including autoimmune diseases, tissue damage, and allograft rejection (Zhao, Yan & Chen, 2013). Macrophages are also recruited to inflammatory microenvironments following attack by pathogenic microorganisms. In addition, as the main target cells for CSFV, infected macrophages facilitated CSFV to gains access to different tissues from the bloodstream (Choi & Chae, 2003). Therefore, in our previous studies, we focused on the interactions between macrophages and CSFV (Ning et al., 2016).

Previous studies have shown that increased expression of AIF1, which boosts the expression of inflammatory mediators and stimulates inflammatory cell proliferation and migration, can be used as a marker of activated macrophages (Zhao, Yan & Chen, 2013). According to a report by Ying Tian et al., inhibition of AIF1 expression induced by siRNA reduces the migration and proliferation of macrophages, and this inhibition of proliferation is not caused by induction of apoptosis (Tian, Kelemen & Autieri, 2006). In another study, it was shown that transfection with AIF1 siRNA can block AIF1 expression, which inhibits inducible nitric oxide synthase (iNOS) production and increases the number of apoptotic macrophages (Zhen et al., 2005). Thus, it is possible that CSFV Shimen affects the survival and migration activities of macrophages by altering the expression of AIF1 to facilitate viral transmission and multiplication.

AIF1 is thought to be involved in various inflammatory diseases, and an early report showed that a widespread upregulation of AIF1 was observed in brains of rats infected with the neurotropic Borna disease virus to characterize the immune-mediated process (Herden, Schluesener & Richt, 2013). However, little is known about its role in the pathogenesis of CSF. In previous study, significantly up-regulated and down-regulated genes were obtained from CSFV Shimen infected group which were compared to the C strain and MOCK infected group (Ning et al., 2017). In this work, we paid attention to AIF1 from the obtained differentially expressed genes to focus on its biological significance in macrophages to CSFV infection. After DGE analysis, we assessed the transcriptional and translational levels of AIF1, which revealed upregulation of AIF1 transcripts and protein in PAMs in response to infection with CSFV Shimen.

It has been well known that activated macrophages produce inflammatory and mitogenic cytokines. The fundamental cellular and molecular events that take place during inflammation are regulated by AIF1, and macrophages produce large amounts of inflammatory cytokines, such as the interleukins IL1 β, IL6, and IL12 (Kimura et al., 2007; Watano et al., 2001). IL6 is often considered to be involved in high fever, such as that experienced by myeloma patients (Murakami et al., 2000). In leukemia patients, IL6 produced by adult T-cell leukemia/lymphoma cells may cause fever and further activate macrophages to secrete TNF-α, leading to shock and exacerbation of the fever (Hiraoka et al., 2010). AIF1 plays a critical role in the regulation of massive synovial proliferation and the production of IL6 in rheumatoid arthritis (Kimura et al., 2007). Thus, we select IL6 as an inflammatory indicator to clarify the role of AIF1 on CSFV Shimen infection in PAMs 3D4/21 cells. As shown in Figs. 1C and 2, CSFV Shimen infection has a significant effect on the transcription and translation of IL6 in macrophages. This is consistent with a previous report by Dong et al., in which the expression of IL6, was greatly increased in CSFV Shimen-infected PAMs (Dong et al., 2013).

The current study has been demonstrated that CSFV infection affected AIF1 and IL6 expression, but whether AIF1 mediate IL6 expression in CSFV infected PAM 3D4/21 cells need to make clear. To this aim, the qPCR analysis was measured to estimate the levels of IL6 in PAM 3D4/21 cells after overexpressing AIF1 treatment. IL6 are known to be an important pro-inflammatory cytokine on variable biological process, and thus some work have reported the recombinant AIF1 can stimulate the production of IL6 in macrophages, such as RAW264.7 cells (Hidetake et al., 2016). This work showed that overexpressed AIF1 stimulated the secretion of IL6 in PAM 3D4/21 cells, which is concordant with previous opinions. When we used siRNA to knockdown AIF1 expression, CSFV Shimen-induced IL6 expression was suppressed. Interestingly, overexpression of AIF1 facilitated CSFV Shimen-induced IL6 expression. Taken together, all these results demonstrated that AIF1 is an important cellular factor that CSFV Shimen triggered AIF1 expression to cause an increase of IL6 expression in PAM 3D4/21 cells.

Viral replication is exactingly relied on host cellular physiological microenvironment. If the AIF1 can affect CSFV Shimen replication remains unknown ago. In the present study, the effect of AIF1 on CSFV Shimen replication in PAM 3D4/21 cells was investigated. Depletion of AIF1 by siRNA inhibited CSFV Shimen growth in PAM 3D4/21 cells while overexpression of recombinant AIF1 was beneficial for the replication of CSFV Shimen. This result suggests that AIF1 has a positive effect on CSFV Shimen infection in macrophages. Many cytokines of host maybe affect CSFV replication (Liang et al., 2016), and we considered AIF1-mediated microenvironment in PAM 3D4/21 cells contributed to CSFV Shimen infection. However, more detailed studies are required to consider this finding, and whether the AIF1 could inhibit other viruses also needs further investigations.

In conclusion, AIF1, which is a highly conserved inflammation-responsive protein, was significantly upregulated in CSFV Shimen-infected **PAM 3D4/21 cells**, and this was accompanied by high IL6 expression, which suggests a possible mechanism for the macrophage-mediated inflammatory response to virulent CSFV infection. This preliminary work demonstrates for the first time the possible role of AIF1 in front of a CSFV Shimen infection, and more information will be expound in future studies.

##  Supplemental Information

10.7717/peerj.8543/supp-1 Figure S1 Bioinformatics analysis of allograft inflammatory factor 1 (AIF1) on spatial structure, phylogenetic tree, and interacting proteins (A) The tertiary structures of AIF1 proteins in various species. The various motifs and domains are represented by different colours. (B) Phylogenetic tree of nine species, drawn using MEGA7.0 software and the neighbour-joining method. (C) Proteins interacting with AIF1 confirmed using STRING 10.5 software. The AIF1 protein is represented in red and the proteins that interact with it are represented in other colours. The thicker lines represent stronger associations. The alignment of the protein sequences is showed in (A) using the BLAST program available from the NCBI website. The AIF1 gene had 93, 91, 91, 90, 90, 61, 61, and 60% identity with the homologous genes in humans, rabbits, sheep, mice, cattle, zebra fish, chickens, and frogs, respectively. A phylogenetic tree was constructed to show the evolutionary relationship between porcine AIF1 and the homologs from other species. Pigs, rabbits, humans, and mice formed a separate clade, which suggests that porcine AIF1 is more closely related to the AIF1 of classical experimental animals.

10.7717/peerj.8543/supp-2 Figure S2 The quantitative analysis to determine the significance of Western blot quantification ImageJ software analysis results showed that AIF1 and IL6 significantly increased after CSFV Shimen infecting in macrophages compared with the mock infection procedure. The results are representative of 3 independent experiments.

10.7717/peerj.8543/supp-3 Figure S3 The quantitative analysis to determine the significance of the confocal results Image analysis results showed that AIF1 (A) and IL6 (B) staining increased after CSFV Shimen infecting in macrophages compared with the mock infection procedure. The results are representative of 3 independent experiments.

10.7717/peerj.8543/supp-4 Figure S4 The mRNA expression of IL6 in macrophages with p-AIF1-NC transfectant qPCR analyzed the expression IL6 mRNA in macrophages with p-AIF1-NC transfectant for 0, 12, 24, and 48 h. The results are representative of 3 independent experiments. “ns” means no significance.

10.7717/peerj.8543/supp-5 Figure S5 Transfection effect and RNA interference on AIF1-p-Si1, AIF1-p-Si2, and AIF1-p-Si3 (A) Fluorescence microscopy was used to observe significant transfection effect after different plasmid groups were carried out plasmid transfection, respectively. (B) The PCR analysis results suggested that AIF1-p-Si1 has a significant inhibitory effect on AIF1 expression compared with the control and AIF1-p-SiNC groups. Lane 1, marker; Lane 2-5, β-actin for AIF1-p-Si1, AIF1-p-Si2, AIF1-p-Si3, AIF1-p-SiNC and the control, respectively; Lane 6-11, AIF1 for AIF1-p-Si1, AIF1-p-Si2, AIF1-p-Si3, AIF1-p-SiNC and the control, respectively.

10.7717/peerj.8543/supp-6 Figure S6 The quantitative analysis to determine the significance of Western blot quantification on AIF1 ImageJ software analysis results showed that a significantly reduced E2 protein expressed of CSFV Shimen was observed in macrophages treated with small interfering RNAs targeting AIF1 whereas a significantly increased E2 protein expressed of CSFV Shimen at 48 h post-infection was observed in macrophages treated with overexpression AIF1 group (p-AIF1). The results are representative of 3 independent experiments.

10.7717/peerj.8543/supp-7 Supplemental Information 1 Uncropped blots (Figs. 2 and 5) Significant results have been obtained though there were nonspecific bands in western blots because this study used Pig cells. Generally, it is difficult to obtain an antibody to pig cells. however, we can use marker to identify the specific band of swine protein.

10.7717/peerj.8543/supp-8 Supplemental Information 2 Raw numeric

## Additional Information and Declarations

### Data Availability

The following information was supplied regarding data availability: The raw measurements are available in the Supplementary Files.
