# Supplementary figures and images for: AIF1 was identified as an up-regulated gene contributing to CSFV Shimen infection in porcine alveolar macrophage 3D4/21 cells
Source: PeerJ. 2020 Feb 17;8:e8543. doi: 10.7717/peerj.8543 (PMC7032059; doi:10.7717/peerj.8543)

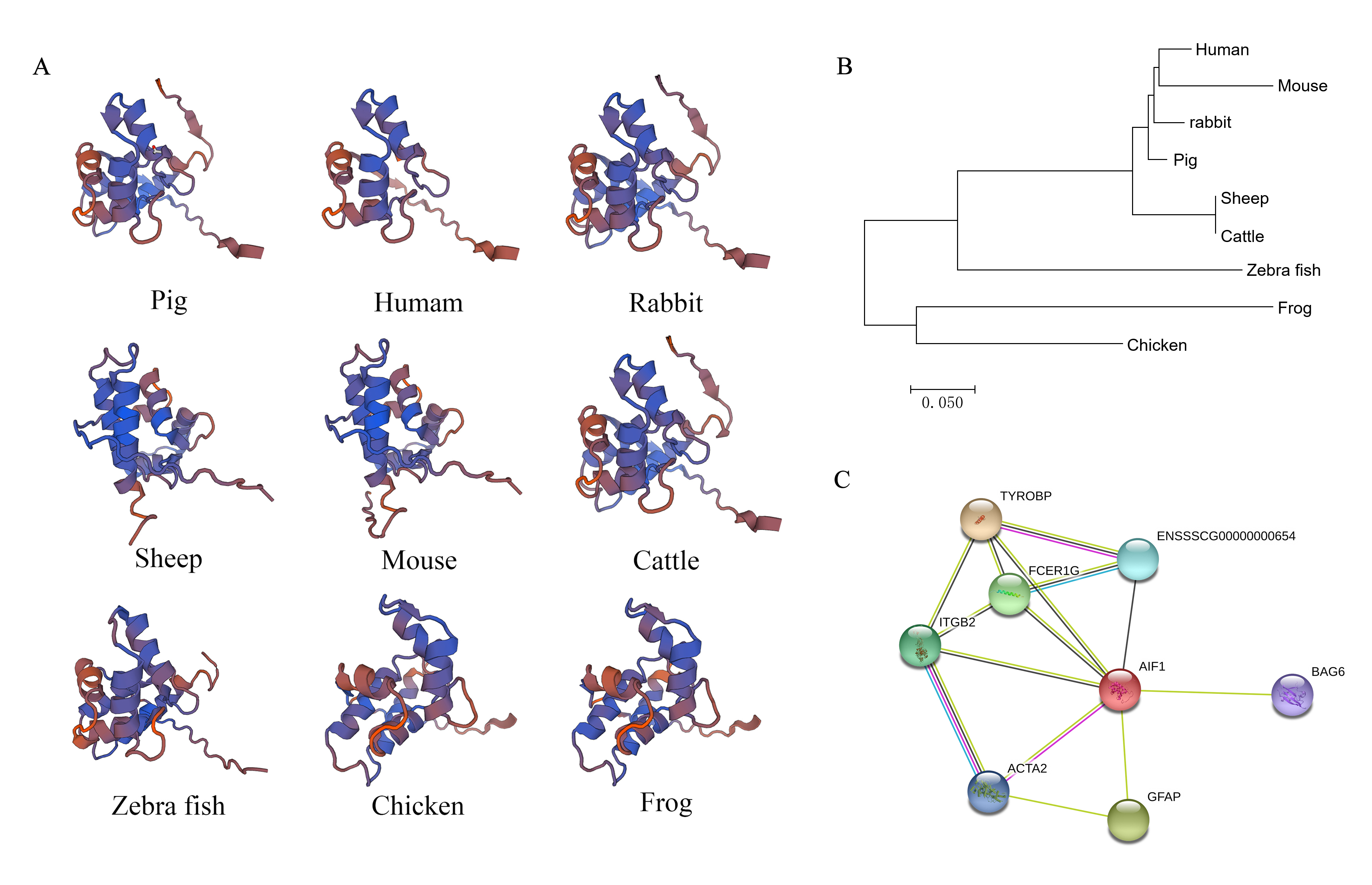

Supplement: Figure S1 — (A) The tertiary structures of AIF1 proteins in various species. The various motifs and domains are represented by different colours. (B) Phylogenetic tree of nine species, drawn using MEGA7.0 software and the neighbour-joining method. (C) Proteins interacting with AIF1 confirmed using STRING 10.5 software. The AIF1 protein is represented in red and the proteins that interact with it are represented in other colours. The thicker lines represent stronger associations. The alignment of the protein sequences is showed in (A) using the BLAST program available from the NCBI website. The AIF1 gene had 93, 91, 91, 90, 90, 61, 61, and 60% identity with the homologous genes in humans, rabbits, sheep, mice, cattle, zebra fish, chickens, and frogs, respectively. A phylogenetic tree was constructed to show the evolutionary relationship between porcine AIF1 and the homologs from other species. Pigs, rabbits, humans, and mice formed a separate clade, which suggests that porcine AIF1 is more closely related to the AIF1 of classical experimental animals. [file peerj-08-8543-s001.jpg]

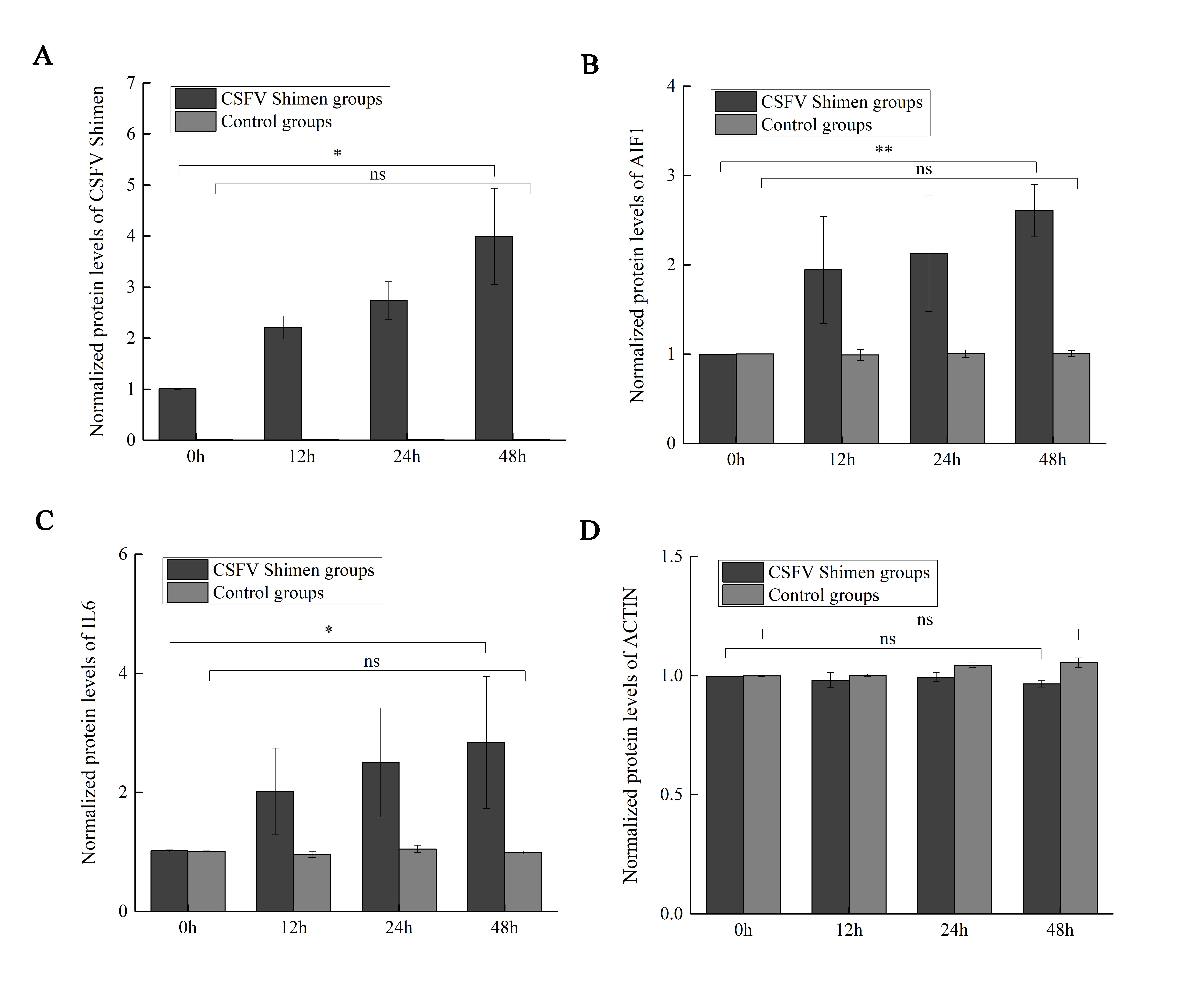

Supplement: Figure S2 — ImageJ software analysis results showed that AIF1 and IL6 significantly increased after CSFV Shimen infecting in macrophages compared with the mock infection procedure. The results are representative of 3 independent experiments. [file peerj-08-8543-s002.jpg]

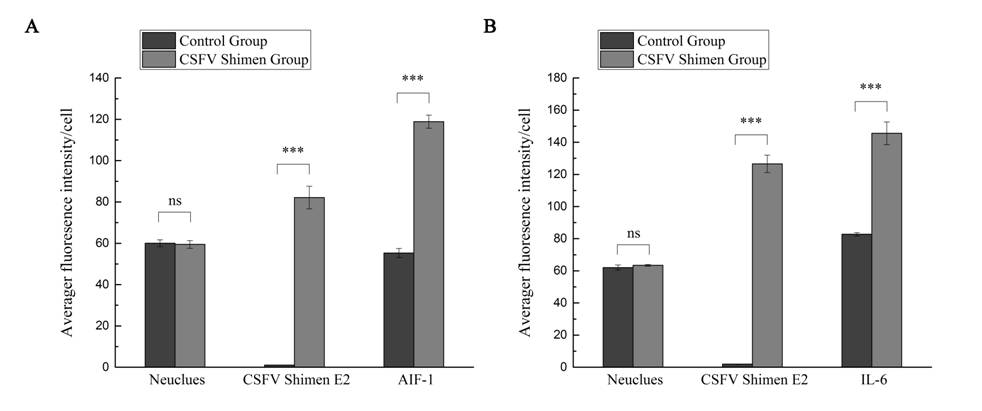

Supplement: Figure S3 — Image analysis results showed that AIF1 (A) and IL6 (B) staining increased after CSFV Shimen infecting in macrophages compared with the mock infection procedure. The results are representative of 3 independent experiments. [file peerj-08-8543-s003.jpg]

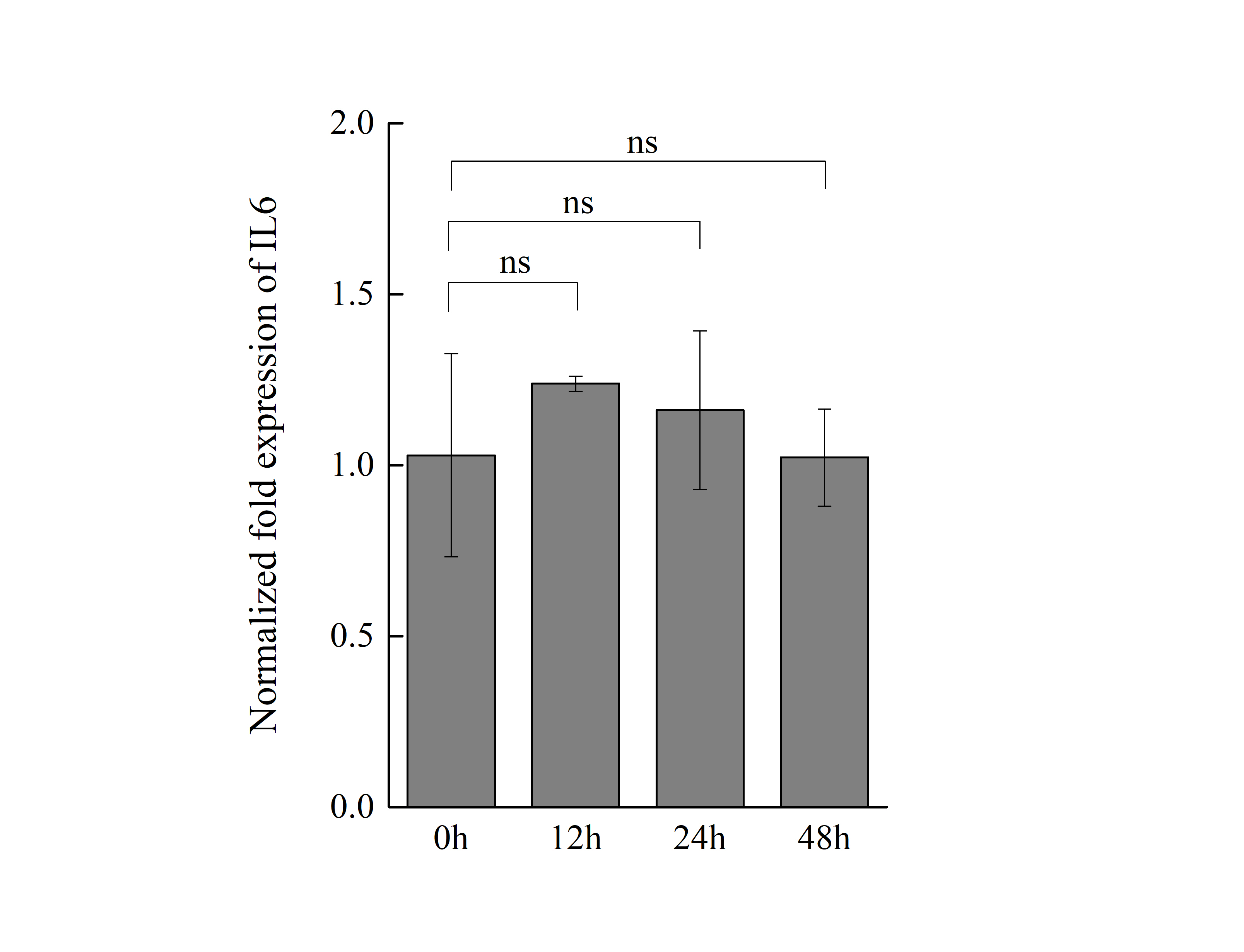

Supplement: Figure S4 — qPCR analyzed the expression IL6 mRNA in macrophages with p-AIF1-NC transfectant for 0, 12, 24, and 48 h. The results are representative of 3 independent experiments. “ns” means no significance. [file peerj-08-8543-s004.jpg]

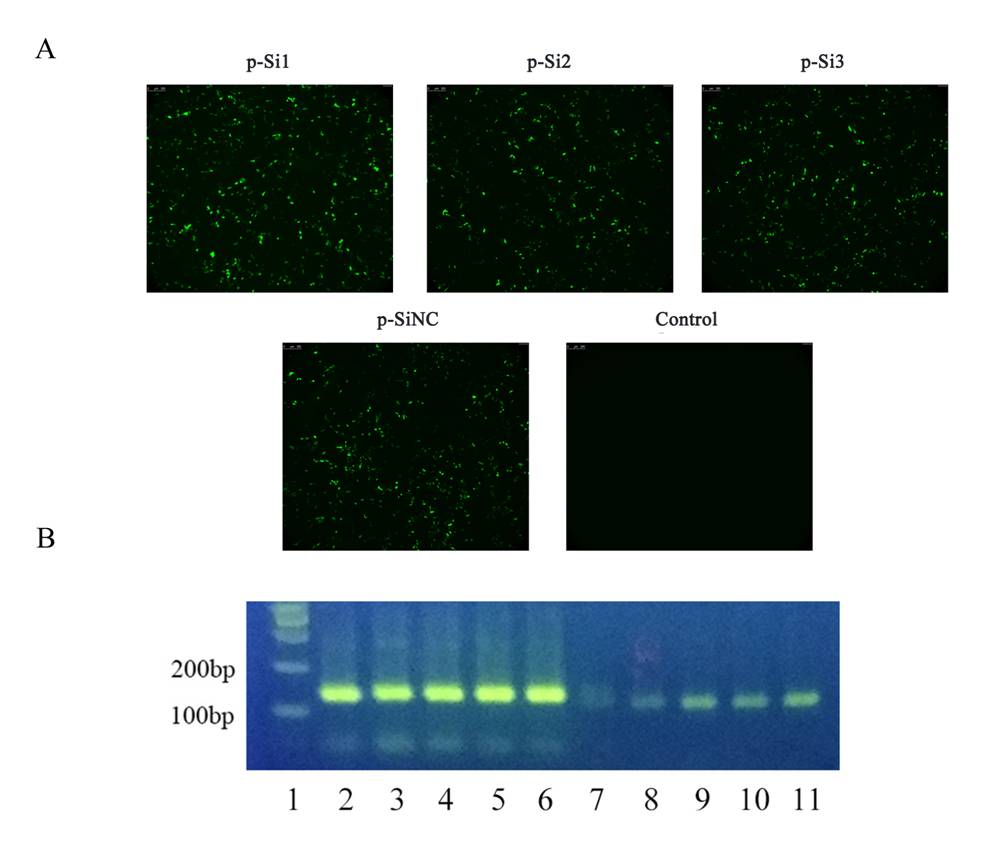

Supplement: Figure S5 — (A) Fluorescence microscopy was used to observe significant transfection effect after different plasmid groups were carried out plasmid transfection, respectively. (B) The PCR analysis results suggested that AIF1-p-Si1 has a significant inhibitory effect on AIF1 expression compared with the control and AIF1-p-SiNC groups. Lane 1, marker; Lane 2-5, β-actin for AIF1-p-Si1, AIF1-p-Si2, AIF1-p-Si3, AIF1-p-SiNC and the control, respectively; Lane 6-11, AIF1 for AIF1-p-Si1, AIF1-p-Si2, AIF1-p-Si3, AIF1-p-SiNC and the control, respectively. [file peerj-08-8543-s005.jpg]

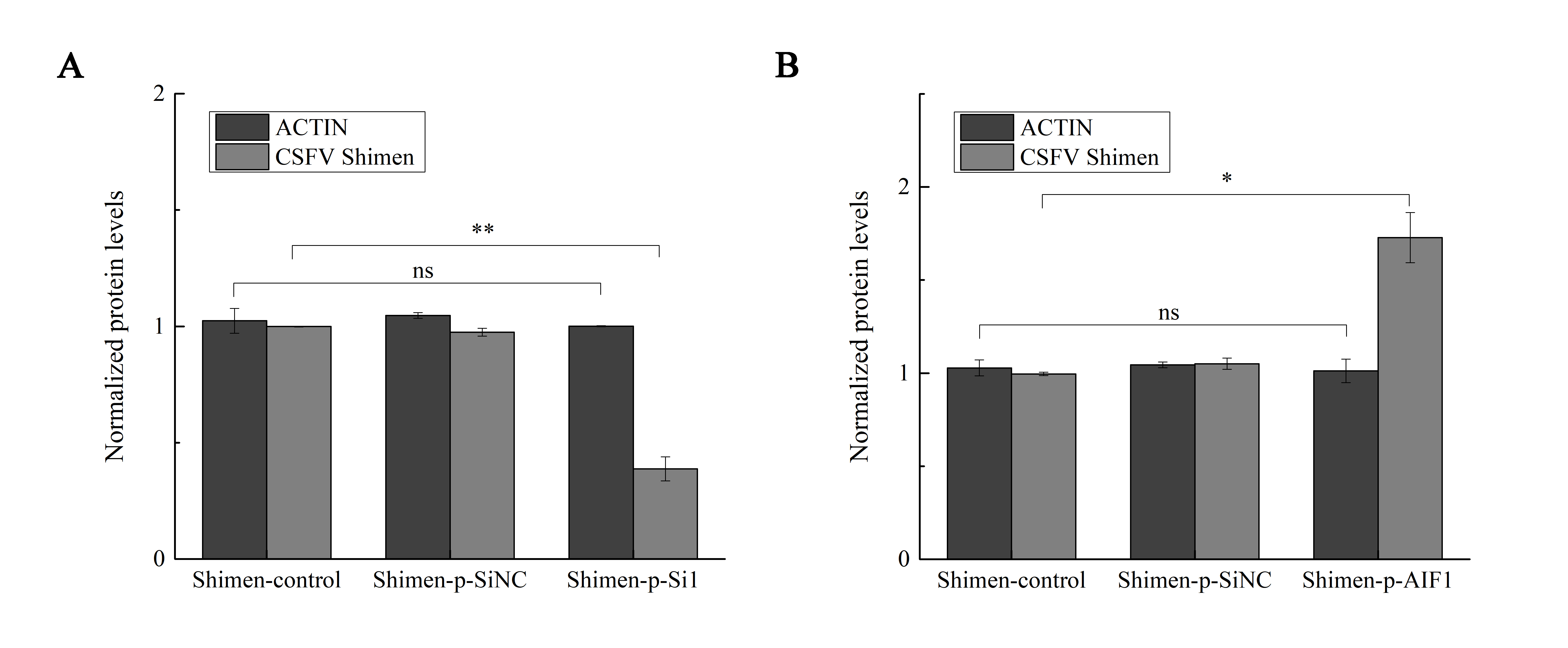

Supplement: Figure S6 — ImageJ software analysis results showed that a significantly reduced E2 protein expressed of CSFV Shimen was observed in macrophages treated with small interfering RNAs targeting AIF1 whereas a significantly increased E2 protein expressed of CSFV Shimen at 48 h post-infection was observed in macrophages treated with overexpression AIF1 group (p-AIF1). The results are representative of 3 independent experiments. [file peerj-08-8543-s006.jpg]
