# Supplementary material for: AIF1 was identified as an up-regulated gene contributing to CSFV Shimen infection in porcine alveolar macrophage 3D4/21 cells
Source: PeerJ. 2020 Feb 17;8:e8543. doi: 10.7717/peerj.8543 (PMC7032059; doi:10.7717/peerj.8543)

Note: Uncropped Figure 2 and Figure 5 were required to unload in submitting system. However, another requisition is the document should be the doc format. The uncropped figures are so many that we list them in this document for facilitate your reviewing.

Uncropped Figure 2 and Figure 5

Figure2A Line 1 -E2


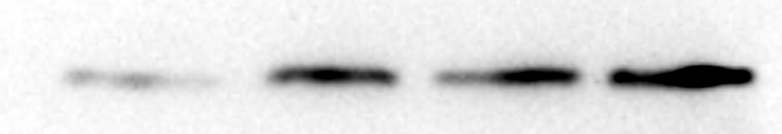


Figure 2A Line 2- AIF1-CSFV SM-signficant results were obtained expelling influence of pig cells to antibodies


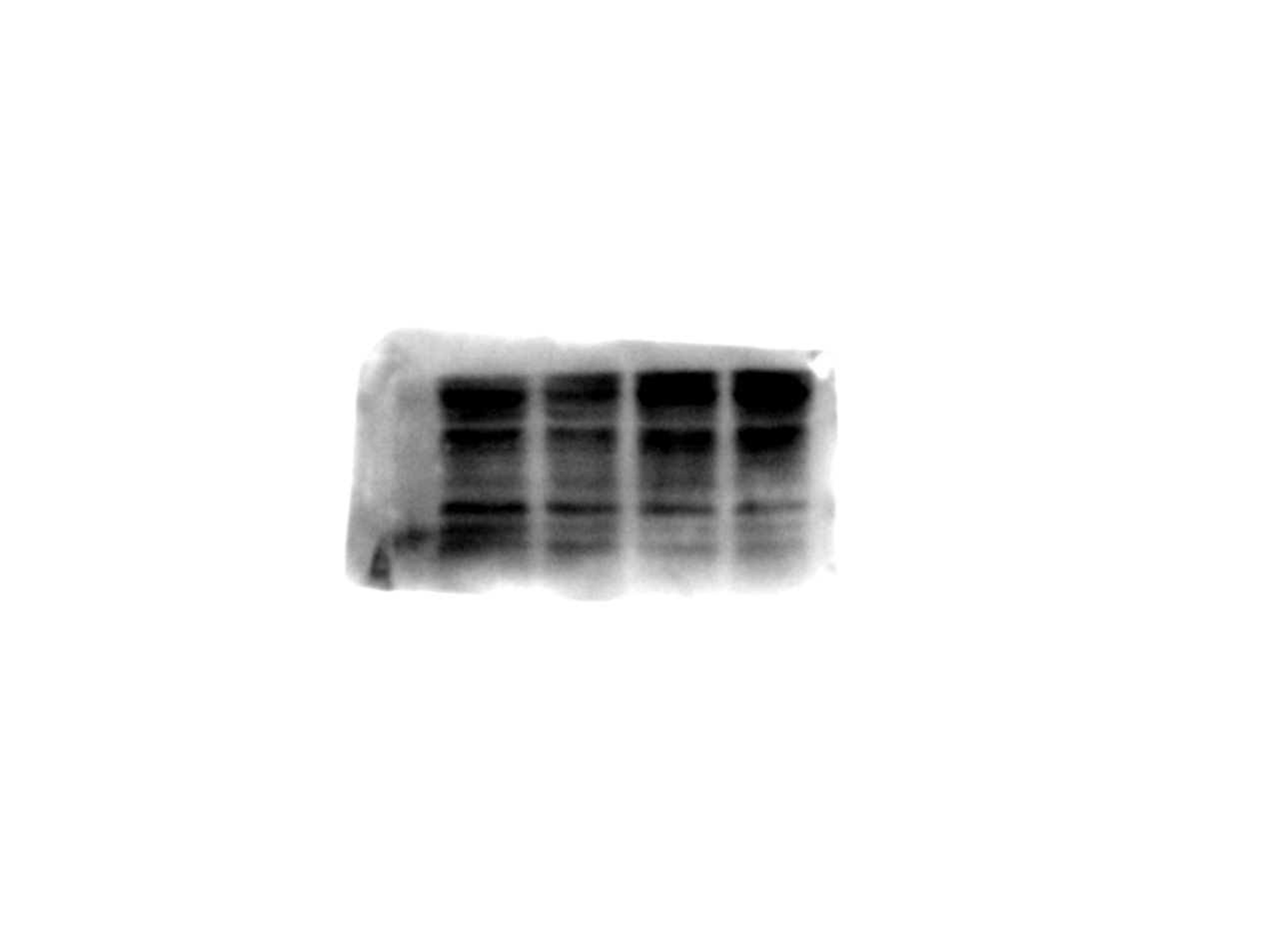


Figure2A line 3 -IL6 for CSFV（right）


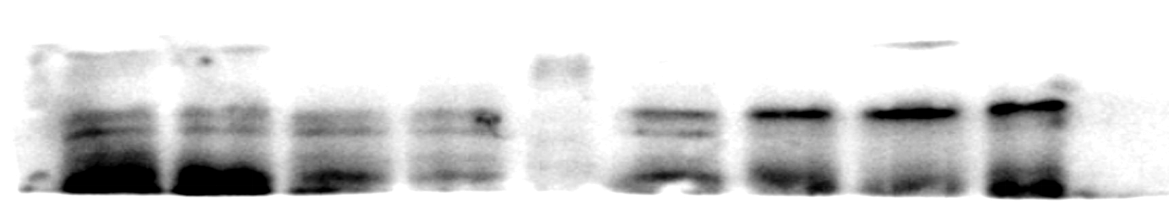


Figure2A line4 -actin –CSFV infection group


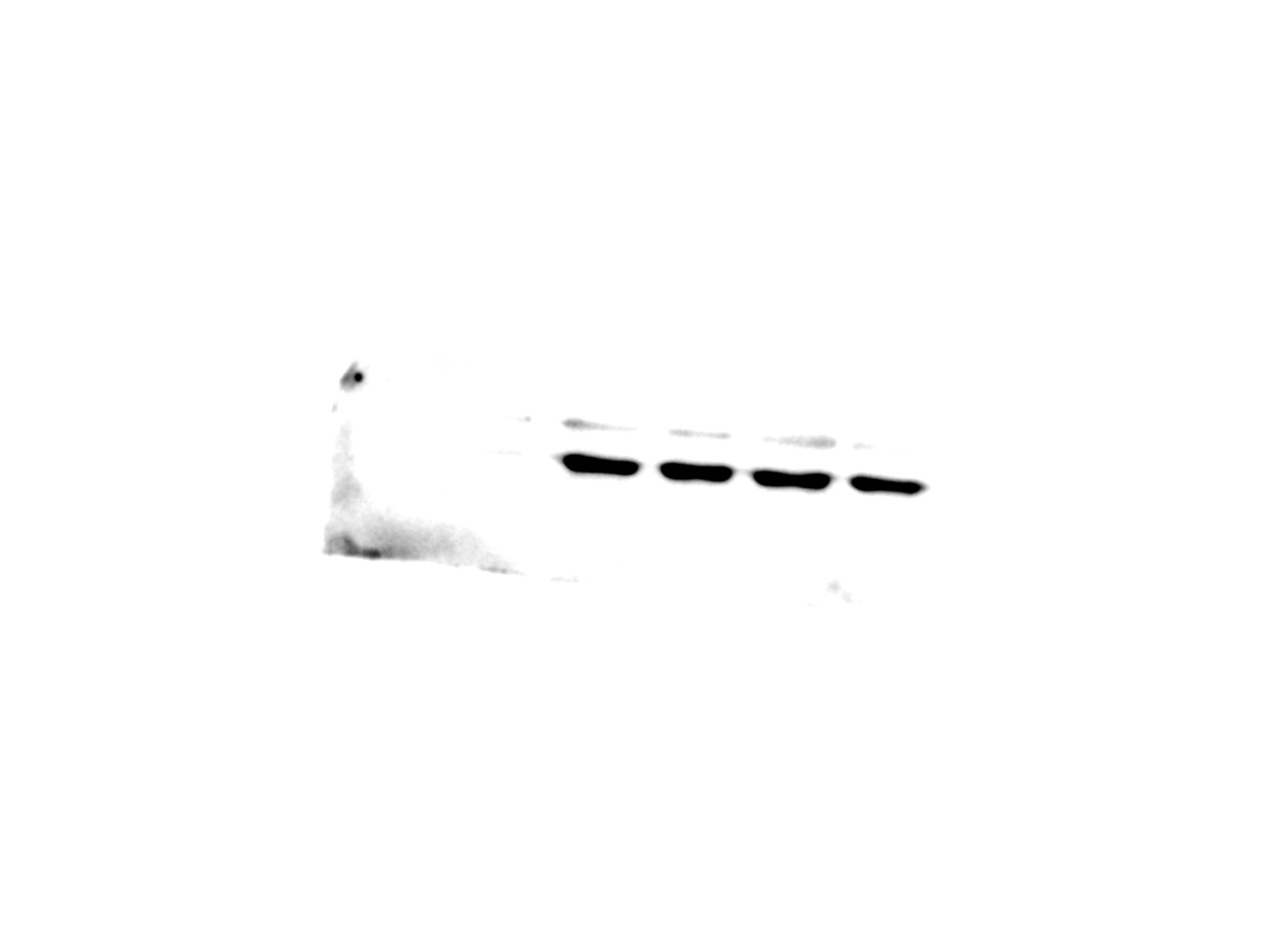


Figure2A line5 - E2-mock infection


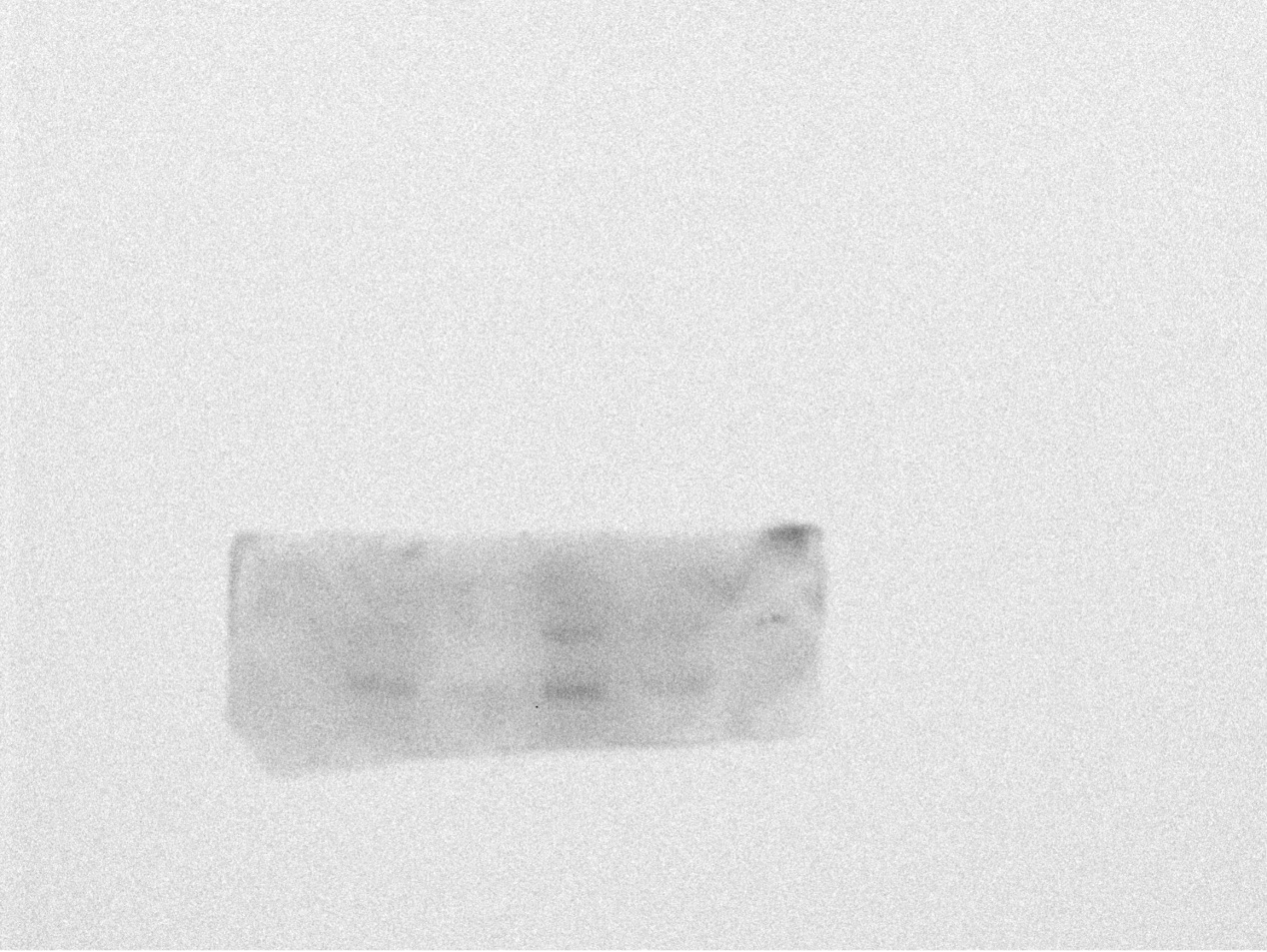


Figure2A line6 AIF-1 from mock infection group


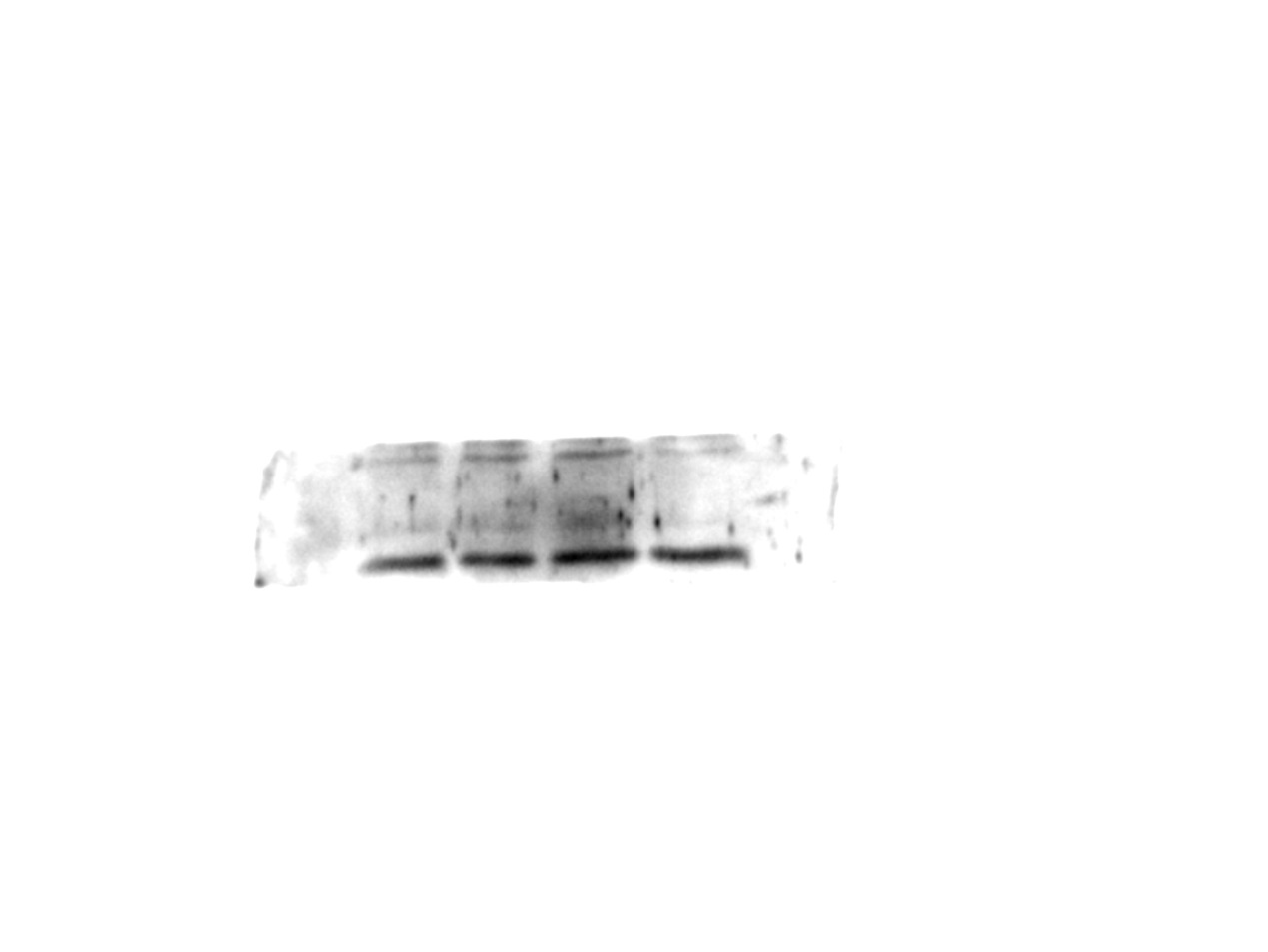


Figure 2A Line7 -IL6 for mock infection（left）


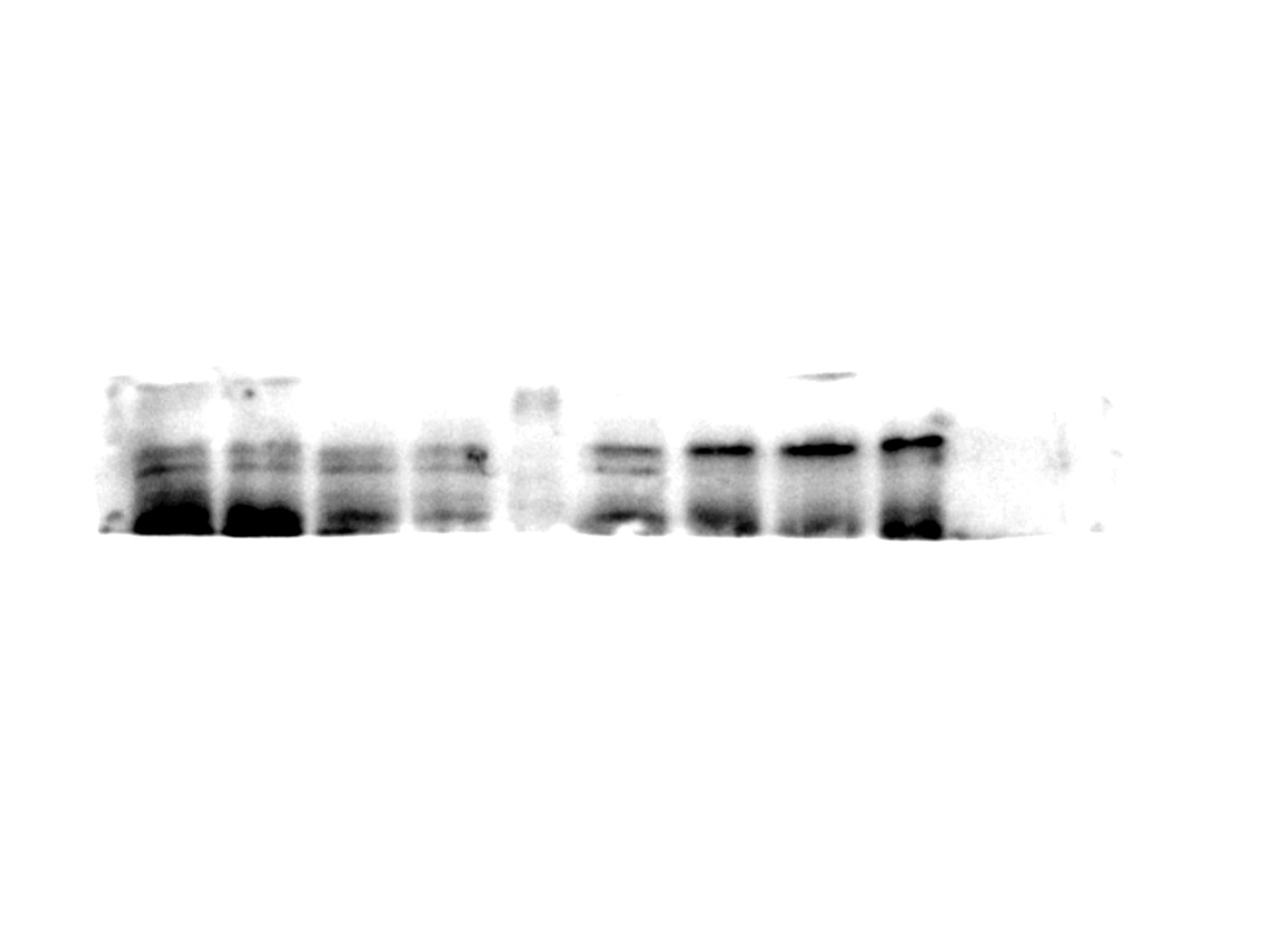


Figure2A line8 -actin –mock infection group


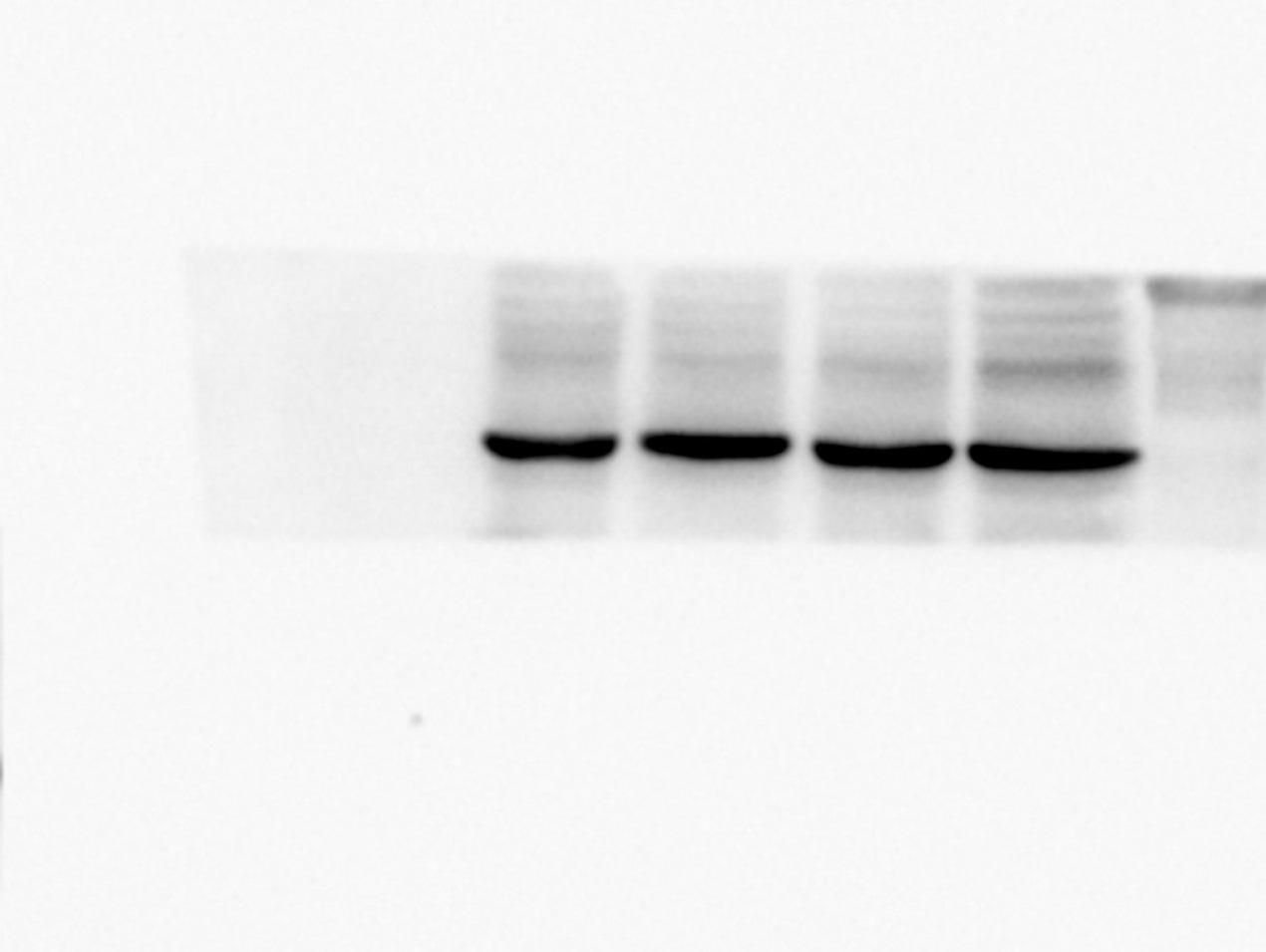


Figure5B line 1（left）actin


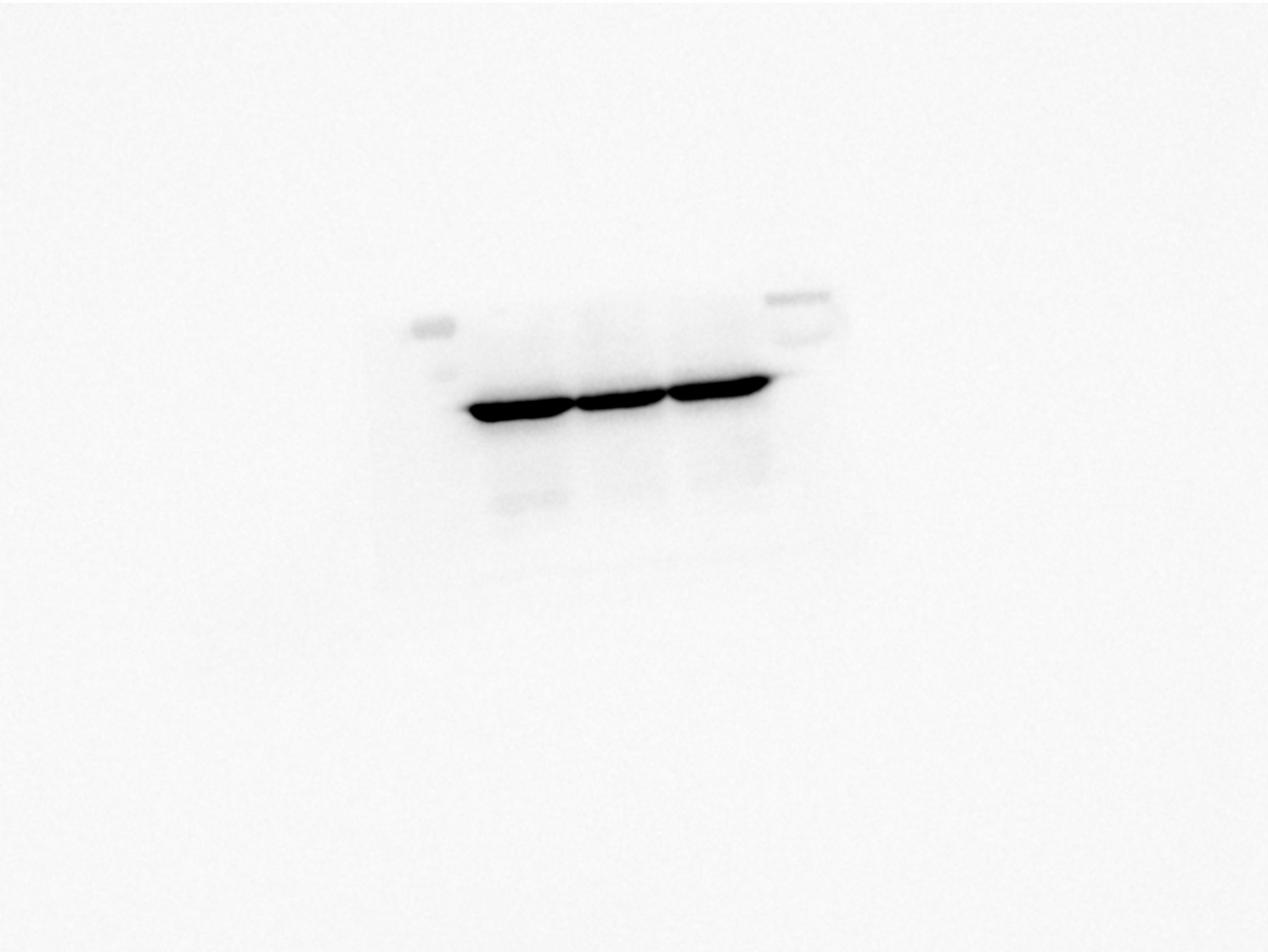


Figure5B line 1（right）actin


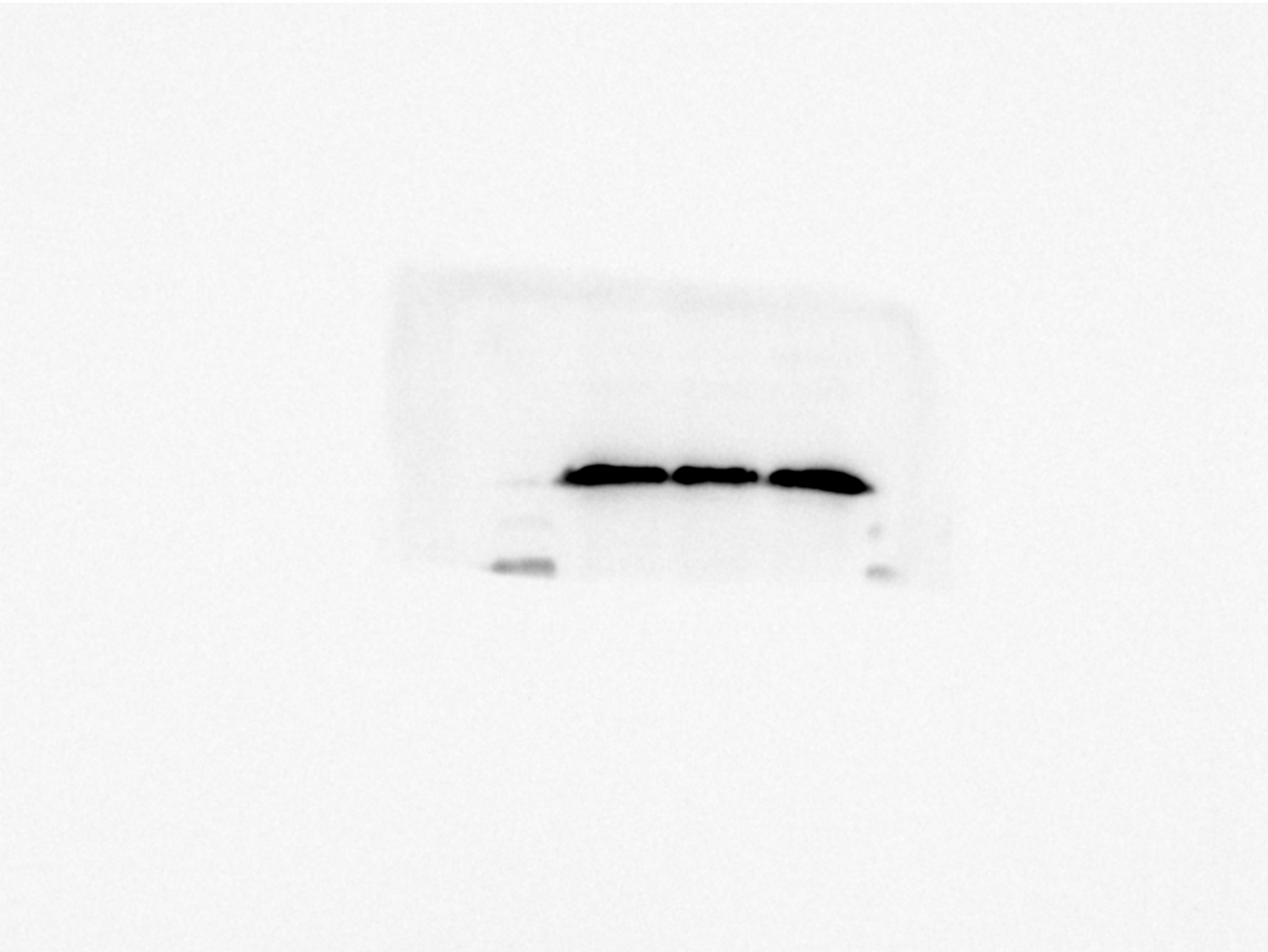


Figure5B line 2（left）E2 for AIF RNAi


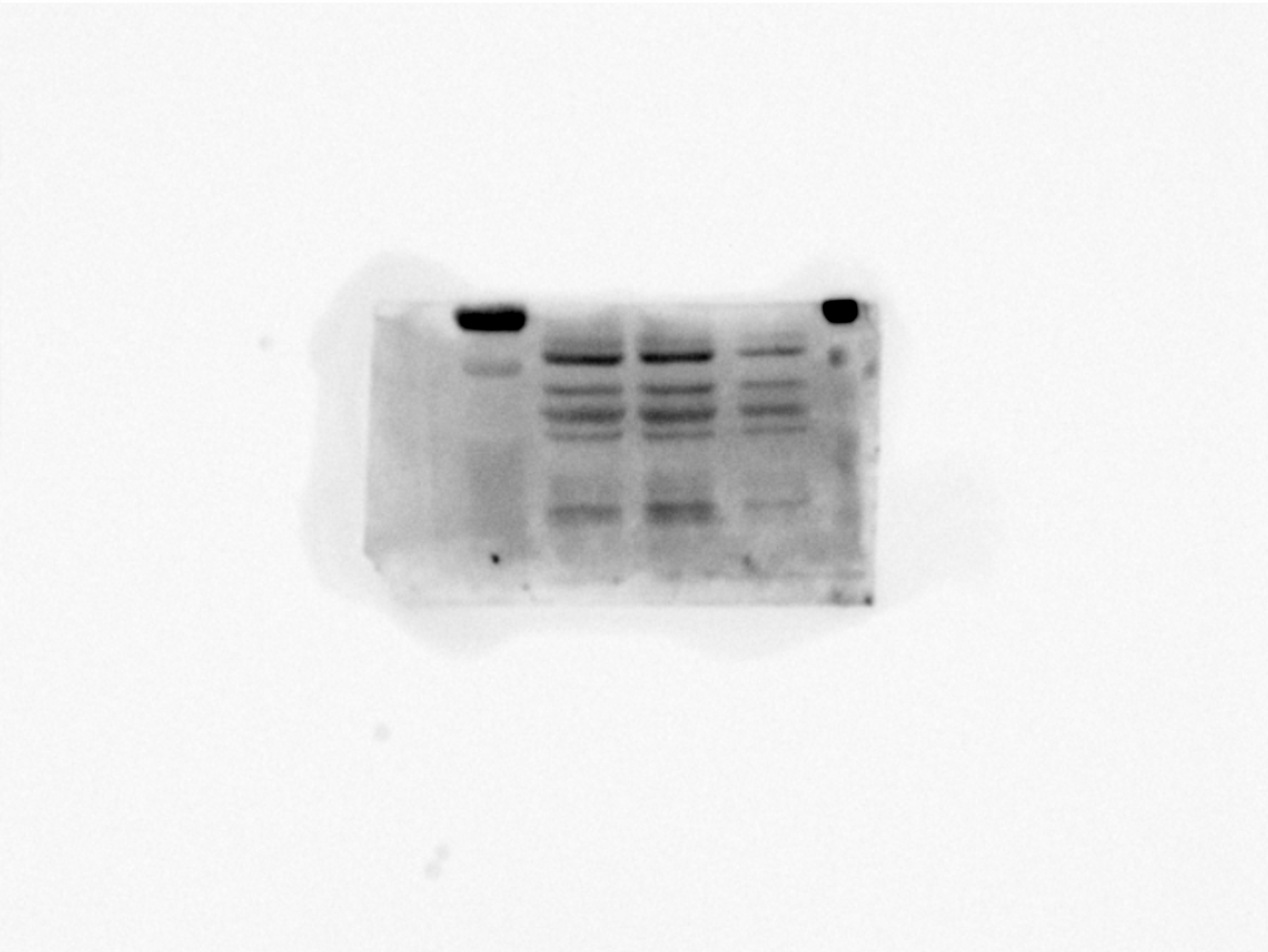


Figure5B line 2（right）E2 for AIF overexpression


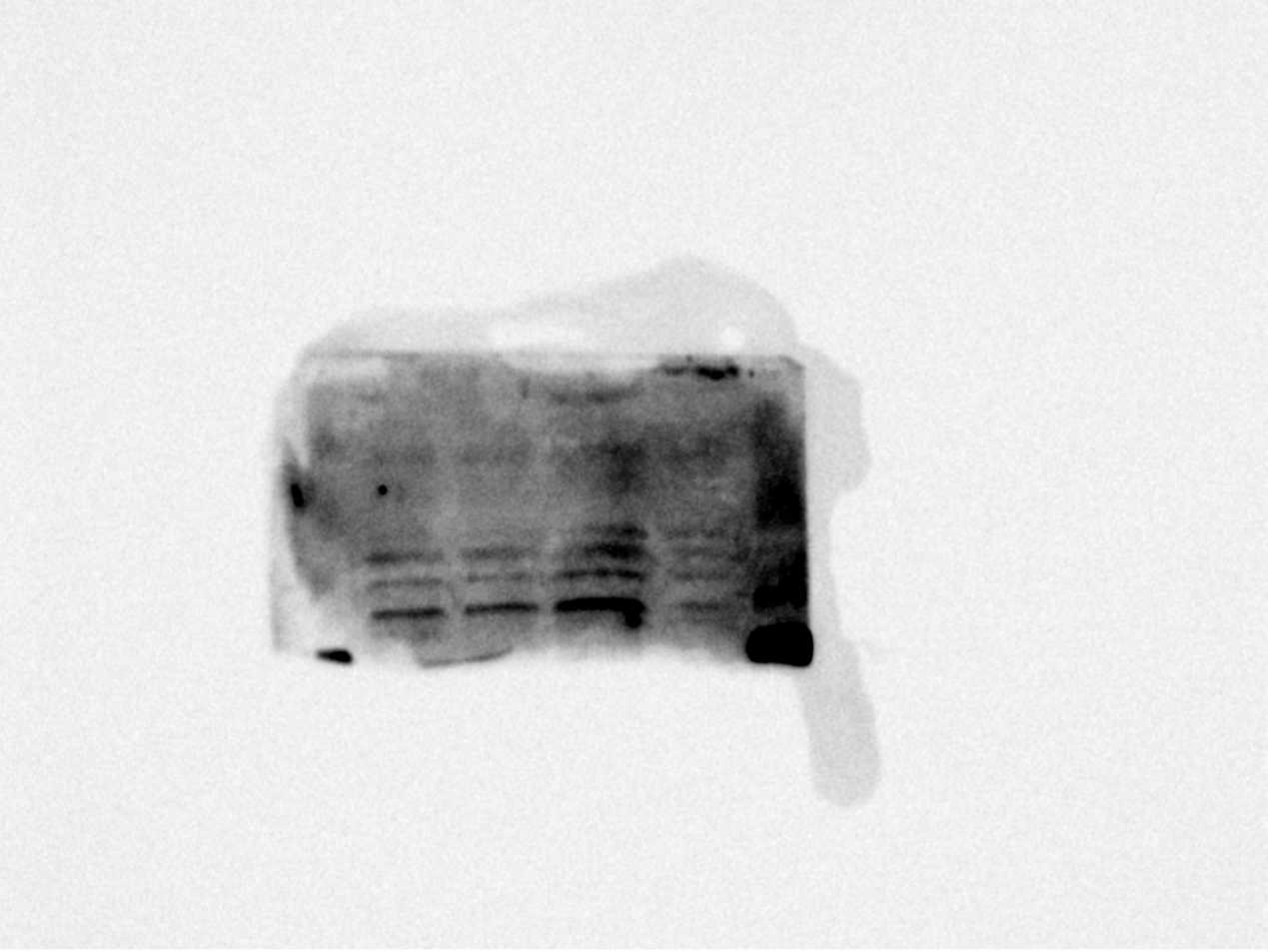

Supplement: Supplemental Information 1 — Significant results have been obtained though there were nonspecific bands in western blots because this study used Pig cells. Generally, it is difficult to obtain an antibody to pig cells. however, we can use marker to identify the specific band of swine protein. [file peerj-08-8543-s007.docx]
